# Identification and knockdown effect of disulfide isomerase in the *Haemaphysalis longicornis* (Acari: Ixodidae)

**DOI:** 10.1590/S1984-29612025058

**Published:** 2025-11-17

**Authors:** Md Samiul Haque, Bumseok Kim, Myung-Jo You

**Affiliations:** 1 Jeonbuk National University, College of Veterinary Medicine and Bio-safety Research Center, Laboratory of Veterinary Parasitology, Iksan-si, Jeollabuk-do, Republic of Korea; 2 Jeonbuk National University, College of Veterinary Medicine, Laboratory of Veterinary Pathology, Iksan-si, Jeollabuk-do, Republic of Korea

**Keywords:** Haemaphysalis longicornis, disulfide isomerase, infection, tick, RNAi, Haemaphysalis longicornis, dissulfeto isomerase, infecção, carrapato, RNAi

## Abstract

Tick-borne diseases are a leading cause of death and illness worldwide. Disulfide isomerase (DSI) is an essential protein that helps tick bodies form natural protein structures so they can perform their biological functions after engorgement. In this study, we are exploring the role of DSI and present a potential strategy for tick management by interfering with DSI in ticks. HlDSI cDNA contains 1,119 nucleotides encoding 372 amino acids, and its molecular weight is 93.69 kilodaltons. BLAST analysis showed that the HlDSI protein was 99.10% identical to DSI-like proteins of other parasites. We identified the Jeju strain of *Haemaphysalis longicornis* and characterized its transcriptional and functional status. Each tick's dsRNA was injected into a female tick and soaked in nymph to further examine its biological functions. RT-PCR and real-time PCR were used to identify and decrease the gene expression of HlDSI RNA interference (RNAi). In adult tick dsRNA-injected groups, HlDSI RNAi significantly impaired tick blood-feeding efficiency and tick viability,and disrupted the molting process in nymphs. According to our analysis, DSI is a significant molecule that is involved in both feeding and reproduction during the tick life cycle,and is therefore a valid target for future tick control strategies.

## Resumo

As doenças transmitidas por carrapatos são uma das principais causas de morte e enfermidades em todo o mundo. A dissulfureto isomerase (DSI) é uma proteína essencial que ajuda os corpos dos carrapatos a formar estruturas proteicas naturais para que possam desempenhar as suas funções biológicas após o ingurgitamento. Neste estudo, exploramos o papel da DSI e apresentamos uma estratégia potencial para a gestão de carrapatos através da interferência com a DSI nos carrapatos. O cDNA do HlDSI contém 1.119 nucleotídeos que codificam 372 aminoácidos e o seu peso molecular é de 93,69 quilodaltons. A análise pelo BLAST mostrou que a proteína HlDSI era 99,10% idêntica às proteínas do tipo DSI de outros parasitos. Identificamos a estirpe Jeju de *Haemaphysalis longicornis* Neumann, 1901 e caracterizamos o seu estado transcricional e funcional. O dsRNA de cada carrapato foi injetado na fêmea e embebido na ninfa para examinar melhor as suas funções biológicas. A RT-PCR e a PCR em tempo real foram utilizadas para identificar e diminuir a expressão genética do HlDSI RNA interference (RNAi). Nos grupos de carrapatos adultos injetados com dsRNA, o RNAi HlDSI prejudicou significativamente a eficiência da alimentação sanguínea e a viabilidade das fêmeas, e perturbou o processo de muda nas ninfas. De acordo com a nossa análise, a DSI é uma molécula importante que está envolvida tanto na alimentação como na reprodução durante o ciclo de vida dos carrapatos, sendo por isso um alvo válido para futuras estratégias de controle.

## Introduction

Ticks are ectoparasitic arthropods that transmit pathogens to both animals and humans globally. Ticks contribute significantly to the global economic decline of cattle production. The three-host tick, *Haemaphysalis longicornis* Neumann, 1901, is common in Asian nations including China, Korea, and Japan and also may be found in Australia and New Zealand (Zhao et al., 2020).

*Haemaphysalis longicornis*, sometimes known as the Asian long-horned tick, carries illnesses including Lyme disease, Babesiosis, and tick-borne encephalitis (Fuente et al., 2008). It is essential for the creation of successful control measures to comprehend the biological mechanisms underpinning tick interactions with hosts. In this study, we investigate whether the absence of disulfide isomerase (DSI) affects the lifecycle of *H. longicornis* ticks.

DSI is an essential enzyme involved in protein folding and maturation, particularly in the formation of disulfide bonds. In arthropods, DSI is crucial for proper development, survival, and immune responses (Tang et al., 2024). The primary structural characteristic of DSI is the presence of two separate thioredoxin domains (Mahmood et al., 2021). However, the role of DSI in tick biology remains poorly understood. The absence of DSI significantly affects the development and survival of *H. longicornis* ticks (Liao et al., 2007). Observations revealed delayed molting, impaired feeding, and reduced engorgement rates in DSI-deficient ticks compared to the control group. These effects were more pronounced during the nymphal and adult stages, indicating a critical role of DSI in tick growth and reproduction (Almazán et al., 2020). Further investigations indicated that DSI deficiency in ticks compromised their immune responses. DSI-deficient ticks exhibited altered expressions of immune-related genes and reduced antimicrobial peptide production, rendering them more susceptible to pathogen infection (Tang et al., 2024). The absence of DSI also influenced the interactions between ticks and their symbiotic microorganisms, potentially disrupting the delicate balance that supports tick survival and pathogen transmission (Di Venere et al., 2015). Additionally, PDI demonstrates chaperone-like function by preventing the aggregation of unfolded proteins irrespective of its catalytic activity (Liu et al., 2017). By preventing the accumulation of unfolded proteins, PDI displays chaperone-like function (Liu et al., 2017). Numerous PDIs have been described from a variety of organisms, including parasites, bacteria, viruses, yeasts, and humans (Knodler et al., 1999; Florenta et al., 2000; Gallina et al., 2002; Kimura et al., 2004). In nematodes, PDI has been shown to be involved in the maintenance of body morphology (Eschenlauer & Page, 2003), larval molting (Chandrashekar et al., 2002), and eggshell collagen formation (Riihimaa et al., 2002). It will be valuable to determine the physiological roles played by DSI in ticks. In 2006, *Amblyomma variegatum* Fabricius, 1794 tick DSI member was identified (Knizetova et al., 2006). Although other pathogen-transmitted vectors like mosquitoes and flies are known to transmit diseases, our understanding of the molecular and biochemical pathways of DSI in ticks is still restricted. The role of DSI in tick biology therefore must be clarified. In this study, we describe the gene discovery, isolation, and sequence analysis of three DSI molecules in *H. longicornis*, and report gene expression patterns according to tick stages. Current studies will be very helpful for further functional analyses of PDI molecules in ticks, as they are involved in molting, important biological functions of ticks, and the conversion of inactive proteins to active proteins.

Here, we report an open reading frame (ORF) of cDNA encoding DSI enzyme in *H. longicornis*. Its transcriptional status was assessed in different life stages and at different tissue levels, and functional characterization of DSI enzyme was also evaluated by RNA interference. Significantly higher expression of DSI enzyme transcription in fed salivary glands and their subsequent gene silencing retarded tick feeding and reproduction. Therefore, targeting salivary DSI enzyme may aid in the future development of tick control strategies.

## Material and Methods

### Ticks and animals

The hard tick *H. longicornis*, specifically the Jeju strain (Tirloni et al., 2015), was cultivated using rabbits at the Laboratory of Veterinary Parasitology, College of Veterinary Medicine and Bio-Safety Research Institute at Jeonbuk National University in Iksan, Republic of Korea. All animals engaged in these experiments were used according to ethical animal research protocols, and their involvement was granted approval by the Animal Care and Use Committee of Jeonbuk National University (Approval code: JBNU 2022-094).

### Immunoscreening of a cDNA expression library

An expressed sequence of the *H. longicornis* DSI gene was identified in a salivary gland cDNA library previously constructed by our laboratory (You et al., 2001). The cDNA library was immune screened by using polyclonal rabbit anti-*H. longicornis* tick immune serum as previously described (You et al., 2001). Pure phage stock was converted to plasmid and the template for sequencing was generated by purification of plasmid DNA using a Plasmid Purification Kit (TaKaRa). An insert cDNA designated DSI gene was sequenced by the dideoxy chain-termination method using M13 reverse and universal primers (Perkin–Elmer, Foster, CA, USA). Sequence analysis was performed using the computer program MacVector (Oxford Molecular, CA, USA).

### Sequence analyses

Nucleotides and deduced amino acid sequences were analyzed using the online EMBOSS translation program (Rice et al., 2000) Multiple sequence alignment was performed using T-coffee (Di Tommaso et al., 2011) combined with Bio Edit software (7.2.1) implementing the Clustal W algorithm using the unknown enolase amino acid sequences of different species from the GenBank database. A phylogenetic tree was constructed with MEGA X software using neighbor-joining (NJ) methods (Kumar et al., 2018).

### Collection of salivary glands

Twenty unfed adult female and male ticks of *H. longicornis* were placed on the ears of specific pathogen-free (SPF) New Zealand White rabbits (Samtako, Korea) using a cloth sock attached with tape. After five days of feeding, partially engorged females were removed for salivary gland collection. Ticks were kept for one hour at room temperature for the removal of host tissue. To prevent surface contamination, the ticks were cleaned with distilled water and 70% ethanol. Salivary glands were collected as described by Patton et al. (2012), but with a minor modification: briefly, ticks were attached to a sterile slide (ventral side down) using liquid paraffin. After that, dissection was performed under a dissecting microscope (SMZ-U; Nikon, Tokyo, Japan) using a scalpel fitted with a no. 11 surgical blade. Salivary glands were separated and washed three times with ice-cold 1× phosphate-buffered saline (PBS) to remove midgut contamination, then stored immediately with RNAlater™ (Ambion, Inc., Austin, TX, USA) at −70 °C.

### Total RNA extraction and synthesis of complementary DNA from tick salivary glands

Total RNA was extracted from the collected salivary glands using a total RNA extraction kit (RiboEx™) in accordance with the manufacturer’s instructions. The concentration of RNA was determined using a NanoDrop™ 2000 spectrophotometer (Thermo Fisher Scientific, Waltham, MA, USA). The sample was then stored at −70 °C. Complementary DNA (cDNA) was synthesized using a transcriptor first-strand cDNA synthesis kit (Roche Holding AG, Basel, Switzerland) in accordance with the manufacturer’s instructions, using 1 µg of total RNA and an anchored oligo (dT)18 primer.

### RT-PCR for detecting DSI

RT-PCR was performed using BioFACT™ 2X Multi-Star PCR Master Mix with a master cycler gradient (Eppendorf, Hamburg, Germany). Primers listed in Table 1 were used for PCR. Actin cDNA was amplified as an internal control using an actin gene-specific primer (Table 1). Primers were designed based on the sequences of DSI and actin accession no AY254898.1, respectively. Amplification was performed using a PCR cycle profile as follows: 95 °C for 15 min, followed by 34 cycles at 95 °C for 20 s, 53 °C for 30 s and 72 °C for one min, with a final extension of five minutes at 72 °C.

**Table 1:** Primers used for identification of disulphide isomerase in *Haemaphysalis longicornis* ticks.

**Primer name**	**5’-3’**	**Product size**
HlDSIF 1	CAAGGATGTGCTGGTCGAGT	590bp
HlDSIR 1	GGGCAAACAACAGATGGCTG	
**DSIF 1**	**AAGGAGGAGTTATGAGGCGG**	**190bp**
**DSIR 1**	**CCCTCTAGATGCATGCTCGA**	
**T7-DSI-F1**	**TAATACGACTCACTATAGGGTACT AAGGAGGAGTTATGAGGCGG**	**190bp**
**T7-DSI-R1**	**TAATACGACTCACTATAGGGTACT CCCTCTAGATGCATGCTCGA**	
**Actin-F1**	**AGCGTGGCTACTCTTTCACC**	**229 bp**
**Actin-R1**	**GATTCCATACCCAGGAACGA**	
**DsF-LacZ-F1**	**GGATCCTAATACGACTCACTATAGGCCCTGGCGTTACCCAACTTA**	
**DsR-LacZ-R1**	**GGATCCTAATACGACTCACTATAGGTCATCCCCGATATGCACCAC**	

The underlined nucleotides indicate T7 region for RNA polymerase binding is in italics.

### Identification of *H. longicornis* DSI genes by sequencing

A plasmid containing putative *H. longicornis* PDI (HlPDI) genes was extracted using the Qiagen DNA Purification kit (QIAGEN, USA). The full-length sequence was obtained by several rounds of sequencing with a plasmid-specific primer and HlPDI gene-specific primers by using the Big Dye terminator method on an ABI PRISM 3100 automated sequencer (Applied Biosystems, Foster City, CA, USA). The amino acid translation of the HlPDI sequences and predicted molecular weight were determined using MacVector software. The alignment and phylogenetic analysis of the sequences were also conducted using MacVector software. The signalP 3.0 Server (Bendtsen et al., 2004) was used to analyze the signal peptide of the genes.

### Synthesis of double-stranded RNA

The PCR products of DSI (190 bp) were joined to a T7 promoter sequence using T7 promoter-linked (at both the 5′ and 3′ ends) oligonucleotide primers (Table 1). A T7 promoter sequence was added as described elsewhere (Bullard et al., 2016) The PCR amplification profile was as follows: 95 °C for 15 min, followed by six cycles at 95 °C for 20 s, 53 °C for 30 s, and 72 °C for one minute, then 28 cycles at 95 °C for 20 s, 77 °C for 30 s, and 72 °C for one minute, with a final extension at 72 °C for five minutes. PCR bands were checked by 1% agarose gel electrophoresis. PCR products were purified using an EZ-Pure™ PCR Purification Kit ver. 2 (Enzynomics, Daejeon, Korea) in accordance with the manufacturer’s instructions. Double-stranded RNA was synthesized from T7 linked DNA using a HiScribe™ T7 High Yield RNA Synthesis Kit (New England Biolabs, Inc., Hitchin, UK) in accordance with the manufacturer’s protocol. The concentration of dsRNA was measured using a NanoDrop™ 2000 spectrophotometer (Thermo Fisher Scientific, Waltham, MA, USA). The sample was then aliquoted and stored at −70 °C until next use.

### Injection of DSI dsRNA double-stranded RNA

Forty adult unfed female ticks were divided into four groups, with each group containing an equal number (10) of ticks. The injection dose for the target gene was selected using methods described in our previous research (Haque et al., 2024a). Two groups of ticks were injected with DSI ds RNA (500 ng/tick), while the negative control (THIRD) group was injected with the DSI LacZdsRNA as a control. Ticks were injected with ds RNA as described elsewhere (Fuente et al., 2006; Kocan et al., 2011) using a Hamilton® 33-gauge needle. After injection, the ticks were kept overnight in a 25 °C incubator with high humidity to observe their survival. The injected female ticks from each group were mixed with an equal number of male ticks and then placed on the ears of four SPF rabbits. After five days of feeding, ten female ticks were taken from each group to observe gene silencing. Subsequently, their salivary glands were collected, and real-time PCR was performed for gene expression analysis. The rest of the ticks were fed with spontaneous drop-downs. Blood engorgement, hatching rate, egg mass weight, and feeding duration were all recorded.

### Nymph soaking with DSI dsRNA

Soaking with liposome-mediated DSI dsRNA was performed as described previously (Zhang et al., 2018). Briefly, 500 μL of liposome mixed DSI dsRNA (1 μg dsRNA/μL liposome) was used, with dsRNA mixed water and liposome at a 1:1 ratio. Control ticks were soaked with the same amount of liposome-mediated unrelated dsRNA (LacZ dsRNA). Ticks (freshly molted adult unfed females and nymphs) were soaked in Eppendorf tubes containing dsRNA at room temperature for 24 h. The ticks were washed with distilled water, dried with tissue paper, and kept in a 25 °C incubator with 95% relative humidity for 3 days before infestation. For infestation, a total of 30 dsRNA-soaked adult female ticks were mixed with an equal number of male ticks and attached to a SPF rabbit ear using cotton bags. Similarly, dsRNA-soaked nymphs were attached to another SPF rabbit ear. From each group, five ticks (adult female and nymph) were collected after three days of attachment and, subsequently, total RNA was extracted for gene silencing analysis. The rest of the ticks were fed to self-drop-down. All ticks were assessed for feeding time, engorgement weight, molting rate, and any abnormalities.

### Analysis of gene silencing at the messenger RNA-level by real-time PCR

Total RNA was collected from the salivary glands of injected female ticks who had fed for five days, as described above. Real-time PCR was performed to determine gene expression after knockdown, using a One-step SYBR® Prime Script™ RT-PCR kit II (Clontech Laboratories, Mountain View, CA, USA) with a Thermal Cycler Dice™ system (Takara, Kyoto, Japan). The primer used for gene expression is cited in Table 1. PCR amplification was carried out in accordance with the manufacturing recommendations. Briefly, PCR amplification was conducted in the following three stages: stage 1 (reverse transcription, 42 °C for five minutes, followed by 95 °C for 10 s); stage 2 (PCR reaction repeats 40 cycles of 95 °C for five seconds, 60 °C for 30 s); and stage 3 (dissociation). Data were normalized with internal control actin and ΔΔCt value, and the percentage of knockdowns was calculated in the same manner as with previous RNAi experiments (Haimes & Kelley, 2010).

### Statistical analysis

Statistical analysis was performed using Student’s t-test (unpaired and unequal variances), executed with Graph Pad Prism 5 (GraphPad Software, Inc., La Jolla, CA, USA). Values were represented in the format of mean ± SE. Statistical significance was defined as p values 0.05 or less when compared to the control group.

## Results

### Sequence analysis of the partial cDNA encoding *H. longicornis* disulphide isomerase

**Figure 1:**
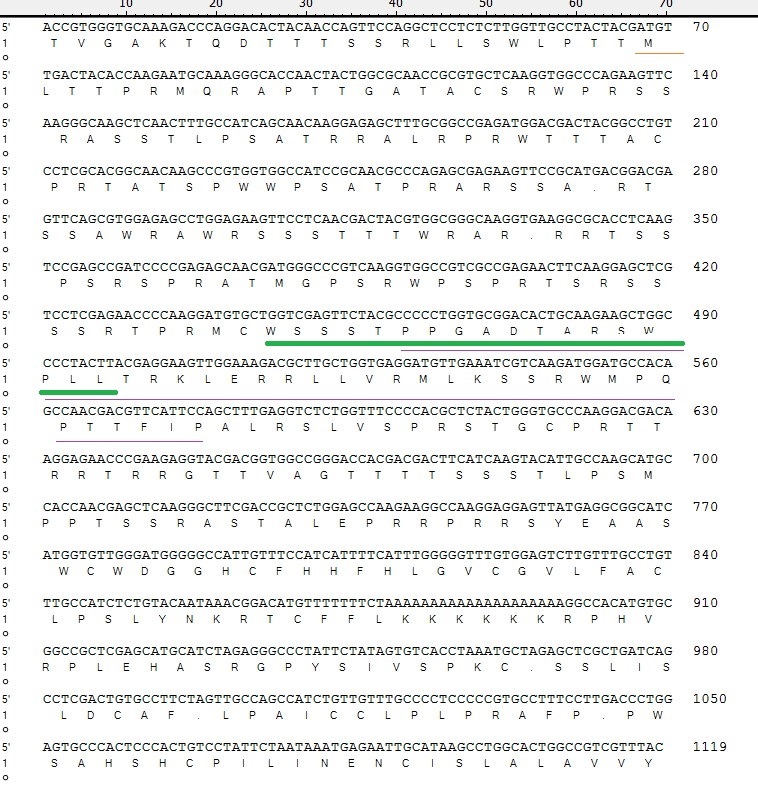
HlDSI nucleotide sequence from *Haemaphysalis longicornis* and its deduced amino acid sequence. The figure highlights the active site region of the protein sequence, spanning amino acid residues 444–498. The sequence of disulfide isomerase contains thioredoxin domain between amino acid positions 127 and 232 (381-696). This domain is functionally and structurally important for enzyme redox activity. The annotation indicates that this region was identified based on Pfam's thioredoxin family profile.

**Figure 2:**
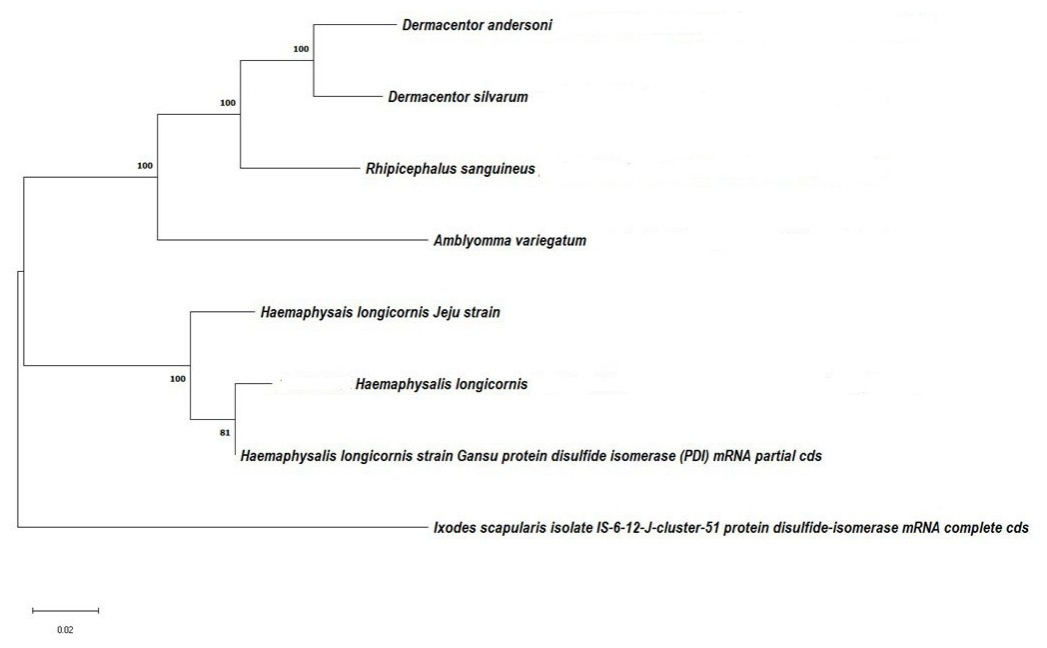
Phylogenetic analysis of DSI from *Haemaphysalis longicornis*. Bootstrap proportions are indicated at branches. Sequences with NJ involve Poisson corrections and bootstrap analysis of 500 replicates.

DSI is a crucial enzyme responsible for phosphorylation of many enzymes in eukaryotic cells. Figure 1 highlights the active site region of the protein sequence, spanning amino acid residues 444–498. The sequence of disulfide isomerase contains the thioredoxin domain between amino acid positions 127 and 232 (381-696). This domain is functionally and structurally important for the enzyme's redox activity. The annotation indicates that this region was identified based on Pfam's thioredoxin family profile (Figure 1). The DSI amino acid sequences were analyzed by NJ with Poisson corrections and 500 bootstrap replicates by MEGA-X software (Figure 2).

### Sequence similarity and phylogenetic analyses

DSI amino acids and nucleotide sequences from different species: *Rhipicephalus sanguineus* (Latreille, 1806 XM_037654809, *A. variegatum* DQ377176, *Dermacentor andersoni* Stiles, 1908 XM_050188851, *H. longicornis* strain Gansu EU016183, *Dermacentor silvarum* Olenev, 1931 XM_037707456, and *Ixodes scapularis* Say, 1821 DQ066336) were collected from the NCBI database and compared for identity with our *H. longicornis* Jeju strain DSI sequence. Identity percentages of DSI nucleotides and amino acids sequences from different species are shown in Figure 2.

### DSI expression profile

**Figure 3:**
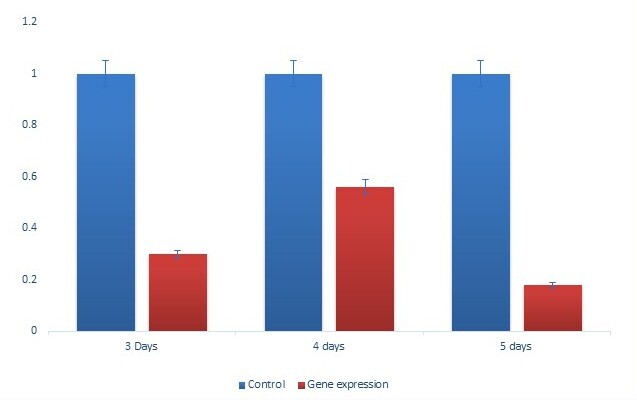
Transcriptional profiles of DSI expression in adult ticks *Haemaphysalis longicornis*.

**Figure 4:**
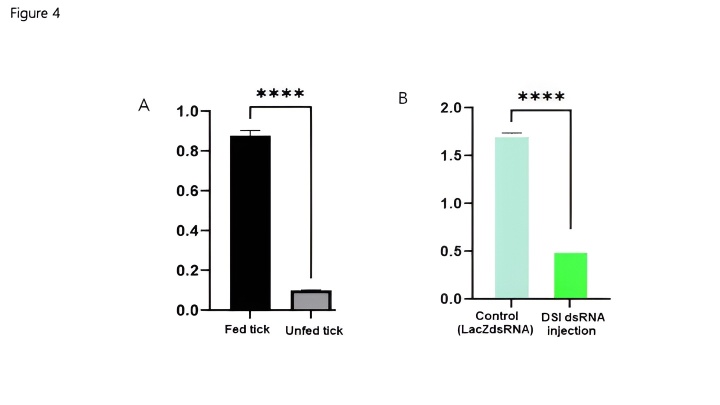
Transcriptional profiles of HlDSI at different developmental stages in ticks *Haemaphysalis longicornis*. (A) DSI expression in unfed versus fed adult ticks. (B) HlDSI expression in DSI injection versus control adult ticks. Significantly different compared to the control group; Data are presented as median ± SD. dsRNA, double stranded RNA. ****p < 0.0001.

HLDSI expression was examined by conventional and real-time PCR (Figures 3-4). These analyses were executed on the third and fourth days of attachment of unfed adult female tick, which were injected with DSIdsRNA or unrelated dsRNA (LacZ dsRNA) and allowed to feed on a rabbit's ear. Different feeding conditions (unfed, fed) in females are presented (Figure 4). HlDSI transcripts were identified on the third and fourth days of attachment (Figure 3) and compared between fed and unfed ticks (Figure 4A). Real-time PCR showed that DSI mRNA was highly expressed on the third day in comparison with the fourth day (Figure 3). Fed ticks without knockdown transcript are higher than those of unfed group (Figure 4B). The expression level of HlDSI mRNA was significantly (*p* < 0.05) higher after blood ingestion, and salivary DSI mRNA expression was also increased as compared with the unfed condition. Adult female ticks (61%) and nymphs (21%) were silenced compared with controls.

### HlDSI knockdown and impacts on attachment, feeding, and reproduction

**Figure 5:**
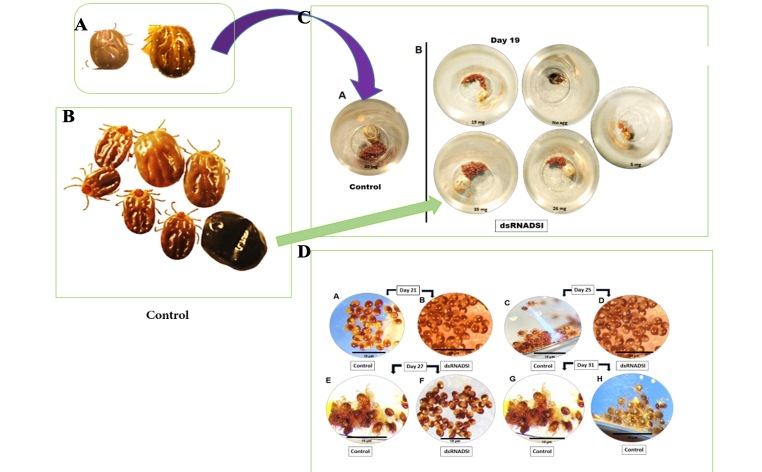
Phenotypic changes in tick (*Haemaphysalis longicornis*) engorgement and egg morphology after DSI dsRNA treatment. (A) Control ticks; (B) DSI dsRNA-treated ticks after spontaneous drop-down; (C) Control tick eggs (A) and eggs of DSI dsRNA-treated ticks after spontaneous drop-down (B); (D) Comparison of embryonic development and hatching of eggs treated with DSI dsRNA compared to control.

For functional analysis of HlDSI in *H. longicornis*, gene silencing thorough RNA interference analysis was executed. Unfed adult female ticks were injected with DSIdsRNA or unrelated dsRNA (LacZ dsRNA) and allowed to feed on a rabbit's ear. Nymphs showed no variation in death rate or attachment rate after soaking with dsRNA compared to control groups. Adult female ticks injected with dsRNA showed significant (*p* < 0.05) variation in feeding duration, engorgement weight, and molting rate compared with control groups, as shown in Table 2. Among adult females injected with HlDSIdsRNA, the average engorgement weight in the treatment group (52.13± 29.73mg/tick) was significantly (*p* < 0.05) lower than that in the control groups (154.33±7.51 mg/tick). Similarly, there was significant (*p* < 0.05) variation in feeding duration at 12 days compared with control at 8 days, as shown in Table 2. However, no variation in attachment rates was observed. To assess the impact of HlDSI on reproduction, egg mass and hatchability were examined after spontaneous dropdown of DSI dsRNA-injected ticks. DSI dsRNA abrogated egg production, and DSI dsRNA-injected ticks showed significantly reduced egg mass (average: 21.25±12.65 mg) compared with the control group (average: 44.66±2.60; Table 2) (*p*<0.05). The egg hatching rate of 31.5% was also significantly reduced (*p*<0.05) compared with that of the control group (Table 2). Phenotypic changes in dsRNA-treated adult female ticks were easily differentiated from those of control ticks (Figure 5A-B). Silenced nymphs showed no variation in death rate or attachment rate after soaking with dsRNA compared to control groups. There was significant (*p* < 0.05) variation in feeding duration, engorgement weight, and molting rate compared with control groups, as shown in Table 3.

**Table 2:** Effects of dsi dsRNA treatment on adult female tick *Haemaphysalis longicornis* blood engorgement^a^.

**Groups**	**Death rate after Injection**	**Attachment rate at 24 h**	**Feeding**	**Engorgement**	**Egg mass**	**Hatching**
			**Duration (days)**	**wt (mg/tick)**	**weight (mg/tick)**	**percentage (%)**
**DSI dsRNA**	**0**	**100**	**12** b	**52.13±29.73^b^**	**21.25±12.65**	**31.5^b^**
**LacZ dsRNA**	**0**	**100**	**8**	**154.33±7.51**	**44.66±2.60**	**80**

a Values are expressed as mean ± standard deviation;

b Significant difference (*P* < 0.05) as calculated by Student's t-test.

**Table 3:** Effects of DSI dsRNA treatment on nymph of *Haemaphysalis longicornis*^a^.

**Groups**	**Death rate after soaking**	**Attachment rate at 24 h**	**Feeding duration (days)**	**Engorgement wt (mg)/10 tick**	**Average days of molting**	**Molting rate (%)**	**Died (%)**
DSI dsRNA	0	98 ± 0.8	7^b^	38± 0.57^b^	15^b^	44^b^	16
LacZ dsRNA	0	99 ± 0.5	5	95± 1.4	21	98	0

a Values are expressed as mean ± standard deviation;

b Significant difference (*p* < 0.05) as calculated by Student's t-test.

### dsRNAi and effects on egg-laying capacity

The percentage of ticks laying eggs was calculated during the oviposition period of control ticks. Most of the engorged ticks (80%) in the Lacz dsRNA-injected group laid eggs, whereas only 31.5% of the engorged ticks in the HlDSI dsRNA-injected group laid eggs and the quantity of eggs was very low (Table 2, Figure 5C) compared to control ticks. Moreover, Table 2 shows that adult female ticks in the treatment groups produced significantly fewer eggs (*p* < 0.05) than the control group. We used 500 eggs in total to observe the incubation period. Hatchability was determined by observing hatching (Figure 5D).

### dsRNAi in nymphs and effects on molting from nymph to adult

**Figure S1:**
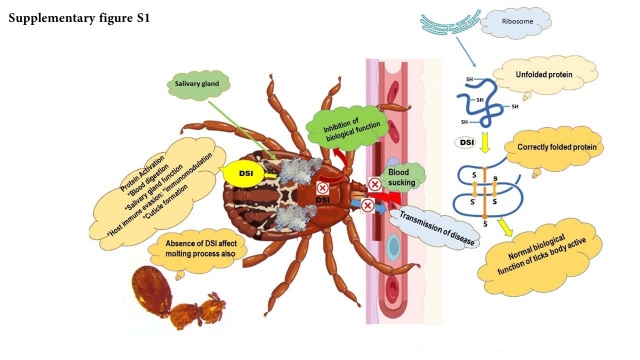
Role of disulfide isomerase (DSI) in various physiological processes of *Haemaphysalis longicornis* ticks.

Disulfide bonds are essential for the correct folding and stability of proteins, and DSI plays roles in their formation and rearrangement. The synthesis and remodeling of the cuticle are significant physiological and biochemical changes that occur during molting. DSI assists in these processes by facilitating the proper folding of cuticle proteins and other structural elements necessary for the formation of the new exoskeleton (Supplementary Material, Figure S1). Moreover, DSI is involved in cellular stress responses, helping to manage oxidative stress generated during this energetically demanding process. These functions underscore the critical role of DSI in enabling successful molting and maintenance. Unfed adult female ticks and nymphs were soaked with DSI dsRNA or unrelated dsRNA (LacZ dsRNA) and allowed to feed on a rabbit's ear. Silencing efficacy was checked by real-time PCR. Successful tick feeding was determined by measuring the attachment rate after 24 h, the engorgement rate, and the molting rate of nymphs and oviposition rate of adult females. The attachment rate was calculated as the percentage of attached ticks against all nymphs, while the engorgement rate was the percentage of engorged ticks compared with control nymphs applied to the ear bag. The molting rate for nymphs was calculated by comparing the number of newly molted adult ticks (Table 2) to the number of all engorged nymphs. Lastly, the rate of oviposition was defined as the number of eggs laying engorged females compared with the number of all engorged female ticks. Among nymph-stage DSIdsRNA soaked ticks, the average engorgement weight in the treatment group (29.75±23.52 mg/tick) was significantly (*p* < 0.05) lower than that of the control groups (207.66±63.2 mg/tick). Similarly, there was significant (p < 0.05) variation in feeding duration compared with control groups, as shown in Table 2. Of the soaked engorged nymphs only 44% molted, while the rest of the HlDSI dsRNA treated ticks died after a blood meal (16%) (Table 2).

## Discussion

The host blood meal is the only source of nutrition and reproduction for the obligatory blood-feeding arthropod *H. longicornis*. The salivary glands of ticks generate a variety of bioactive compounds during blood intake, which aid in the tick's blood engorgement and facilitate the spread of disease. To our knowledge, there are no data related to the role of HlDSI in tick-host interference. Therefore, this study was carried out to identify and characterize DSI in the *H. longicornis* Jeju strain. Through gene silencing, we analyzed the functional significance of DSI in feeding (adult and nymph) and reproduction of *H. longicornis*, the most common tick species in Korea (Lee et al., 2004; Chae et al., 2019).

We investigated the HlDSI gene and explored its potential roles during attachment, blood feeding, and reproduction in adult ticks and molting in nymphs. Through RNAi knockdown of DSI, we found that DSI impairs attachment, blood feeding, and reproduction in adult ticks, and molting in nymphs. Through gene silencing, we analyzed the role of DSI in feeding and reproduction of *H. longicornis*. Our results indicate that PDI genes are important for tick biology, especially for egg development, and that they play distinct roles in different tissues. In a previous study, blood feeding induced significantly increased expression of HlPDI-1 and HlPDI-3 in both partially fed nymphs and adults (Liao et al., 2007). Recently, Sialo transcriptome analysis of *H. longicornis* carried out by our research group (Tirloni et al., 2015) revealed the presence of significant amounts of an DSI-like homologous protein in salivary fluid in both adult and nymph stages. Together, these findings indicate that DSI is a secretory protein that plays an important role in successful tick feeding. DSI can ensure that proteins are correctly folded in the ER by catalyzing the formation and breaking of disulfide bonds between cysteine residues within proteins.

DSIs have been identified from other tick species, including *A. variegatum* (Knizetova et al., 2006) and are known to be triggered in blood feeding, cuticle formation, and oviposition. DSI like molecules are a common feature amongst all eukaryotes studied. In yeast, the genes encoding for six PDI-like molecules have been implicated in native disulfide bond formation (Frand & Kaiser, 2000). Nine PDI-like molecules from five species of the malaria parasite *Plasmodium* have also been reported (Mahajan et al., 2006).

In this study, PDI molecules were isolated using cDNA libraries of *H. longicornis*. This is the first report of more than one DSI member in a tick species. Through gene silencing, we analyzed the role of DSI in feeding and reproduction of H. longicornis. DSI were previously shown to be significantly up-regulated in blood-feeding nymphs and adults as well as in B. gibsoni-infected larvae (Essa et al., 2024).

**Figure 6:**
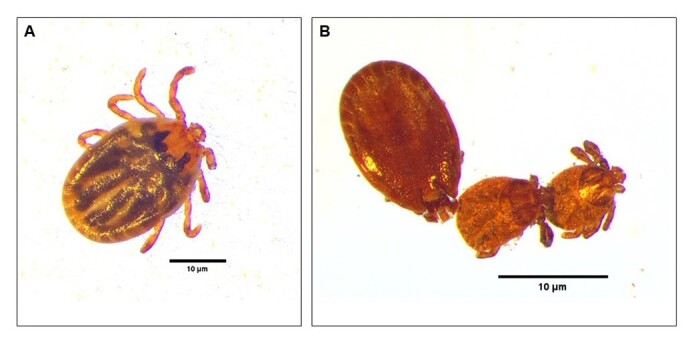
Effects of DSI dsRNA on molting from nymph to adult of *Haemaphysalis longicornis*.

RNAi has become the most widely used gene-silencing technique (Fuente et al., 2007) and has recently been used for the analysis of gene function in ticks. We applied RNAi in a previous study (Haque et al., 2024b) to determine the functional role of DSI. RNA interference acts by degrading targeted mRNA as a result of specific gene inhibition. Injection of DSI dsRNA in ticks caused silencing of DSI expression at the mRNA level in 61% of adults and 21% of nymphs compared with control groups. DSI knockdown was found to have major impacts on blood engorgement, abnormal egg production, and hatchability (Figure 5). Our results show that when dsRNAs of HlDSI genes are individually introduced into ticks they resulted in phenotypic changes (Figure 5). In the HlDSI dsRNA-injected group, the body weight of the engorged ticks was significantly reduced and the engorged ticks laid few or no eggs (Figure 5). Nymphs displayed delayed or unsuccessful molting (Figure 6), which results in developmental defects. Taken together, these results suggest that DSI has important roles in embryonic development (Figure 5).

To our knowledge this is first report describing the molecular characterization of DSI in the Jeju strain of H. longicornis. Silencing of HlDSI affects the feeding and reproductive capabilities of ticks. Further studies are required for recombinant HlDSI production and assessment of its potential as a vaccine candidate for the control of tick populations (Han et al., 2014). This study highlights the vital role that DSI plays in the life cycle of *H. longicornis,* impacting immune responses, development, survival, and interactions with pathogens. The absence of DSI disrupts key biological processes, highlighting its essential function in tick physiology. Given the significance of disulfide bonds in structural and functional protein stability, targeting DSI presents a promising strategy for tick control.

## Supplementary Material

This material is available as part of the online article from https://doi.org/10.1590/S1984-29612025058.

## References

[B001] Almazán C, Fourniol L, Rakotobe S, Šimo L, Bornères J, Cote M (2020). Failed disruption of tick feeding, viability, and molting after immunization of mice and sheep with recombinant *Ixodes ricinus* salivary proteins IrSPI and IrLip1. Vaccines.

[B002] Bullard RL, Williams J, Karim S (2016). Temporal gene expression analysis and RNA silencing of single and multiple members of gene family in the lone star tick *Amblyomma americanum.*. PLoS One.

[B003] Bendtsen JD, Nielsen H, von Heijne G, Brunak S (2004). Improved prediction of signal peptides: SignalP 3.0. J Mol Biol.

[B004] Chae JB, Cho YS, Cho YK, Kang JG, Shin NS, Chae JS (2019). Epidemiological investigation of tick species from near domestic animal farms and cattle, goat, and wild boar in Korea. Korean J Parasitol.

[B005] Chandrashekar R, Devarajan E, Mehta K (2002). *Dirofilaria immitis*: further characterization of the transglutaminase enzyme and its role in larval molting. Parasitol Res.

[B006] Di Venere M, Fumagalli M, Cafiso A, De Marco L, Epis S, Plantard O (2015). *Ixodes ricinus* and Its Endosymbiont *Midichloria mitochondrii*: a comparative proteomic analysis of salivary glands and ovaries. PLoS One.

[B007] Di Tommaso P, Moretti S, Xenarios I, Orobitg M, Montanyola A, Chang JM (2011). T-Coffee: a web server for the multiple sequence alignment of protein and RNA sequences using structural information and homology extension. Nucleic Acids Res.

[B008] Eschenlauer SCP, Page AP (2003). The *Caenorhabditis elegans* ERp60 homolog protein disulfide isomerase-3 has disulfide isomerase and transglutaminase-like cross-linking activity and is involved in the maintenance of body morphology. J Biol Chem.

[B009] Essa IM, Azzal GY, Thamer NK (2024). First molecular sequencing of *Babesia gibsoni* in ticks, Iraq. Open Vet J.

[B010] Florenta I, Mouray E, Dali Ali F, Drobecq H, Girault S, Schrével J (2000). Cloning of *Plasmodium falciparum* protein disulfide isomerase homologue by affinity purification using the antiplasmodial inhibitor 1,4-bis[3-[N-(cyclohexyl methyl)amino]propyl]piperazine. FEBS Lett.

[B011] Frand AR, Kaiser CA (2000). Two pairs of conserved cysteines are required for the oxidative activity of Ero1p in protein disulfide bond formation in the endoplasmic reticulum. Mol Biol Cell.

[B012] Fuente J, Almazán C, Naranjo V, Blouin EF, Kocan KM (2006). Synergistic effect of silencing the expression of tick protective antigens 4D8 and Rs86 in *Rhipicephalus sanguineus* by RNA interference. Parasitol Res.

[B013] Fuente J, Kocan KM, Almazán C, Blouin EF (2007). RNA interference for the study and genetic manipulation of ticks. Trends Parasitol.

[B014] Fuente J, Estrada-Pena A, Venzal JM, Kocan KM, Sonenshine DE (2008). Overview: ticks as vectors of pathogens that cause disease in humans and animals. Front Biosci.

[B015] Gallina A, Hanley TM, Mandel R, Trahey M, Broder CC, Viglianti GA (2002). Inhibitors of protein-disulfide isomerase prevent cleavage of disulfide bonds in receptor-bound glycoprotein 120 and prevent HIV-1 entry. J Biol Chem.

[B016] Haimes J, Kelley M (2010). Demonstration of a ΔΔ C q calculation method to compute relative gene expression from qPCR data..

[B017] Han H, Dong H, Zhu S, Zhao Q, Jiang L, Wang Y (2014). Molecular characterization and analysis of a novel protein disulfide isomerase-like protein of *Eimeria tenella.*. PLoS One.

[B018] Haque MS, Islam MS, You MJ (2024). Effect of Silencing subolesin and enolase impairs gene expression, engorgement and reproduction in *Haemaphysalis longicornis* (Acari: Ixodidae) ticks. J Vet Sci.

[B019] Haque MS, Rahman MK, Islam MS, You MJ (2024). Molecular cloning, identification, transcriptional analysis, and silencing of enolase on the life cycle of *Haemaphysalis longicornis* (Acari, Ixodidae) tick. Parasites Hosts Dis.

[B020] Kimura T, Hosoda Y, Kitamura Y, Nakamura H, Horibe T, Kikuchi M (2004). Functional differences between human and yeast protein disulfide isomerase family proteins. Biochem Biophys Res Commun.

[B021] Knizetova P, Vancova I, Kocakova P, Slovak M, Proost P, Kopacek J (2006). New member of the protein disulfide isomerase (PDI) family identified in *Amblyomma variegatum* tick. Insect Biochem Mol Biol.

[B022] Knodler LA, Noiva R, Mehta K, McCaffery JM, Aley SB, Svärd SG (1999). Novel protein-disulfide isomerases from the early-diverging protist *Giardia lamblia.*. J Biol Chem.

[B023] Kocan KM, Blouin E, Fuente J (2011). RNA interference in ticks. J Vis Exp.

[B024] Kumar S, Stecher G, Li M, Knyaz C, Tamura K (2018). MEGA X: Molecular Evolutionary Genetics Analysis across Computing Platforms. Mol Biol Evol.

[B025] Lee JH, Park HS, Jang WJ, Koh SE, Park TK, Kang SS (2004). Identification of the *Coxiella* sp. detected from *Haemaphysalis longicornis* ticks in Korea. Microbiol Immunol.

[B026] Liao M, Hatta T, Umemiya R, Huang P, Jia H, Gong H (2007). Identification of three protein disulfide isomerase members from *Haemaphysalis longicornis* tick. Insect Biochem Mol Biol.

[B027] Liu G, Wang J, Hou Y, Huang Y, Li C, Li L (2017). Improvements of modified wheat protein disulfide isomerases with chaperone activity only on the processing quality of flour. Food Bioprocess Technol.

[B028] Mahajan B, Noiva R, Yadava A, Zheng H, Majam V, Mohan KV (2006). Protein disulfide isomerase assisted protein folding in malaria parasites. Int J Parasitol.

[B029] Mahmood F, Xu R, Awan MUN, Song Y, Han Q, Xia X (2021). PDIA3: Structure, functions and its potential role in viral infections. Biomed Pharmacother.

[B030] Patton TG, Dietrich G, Brandt K, Dolan MC, Piesman J, Gilmore RD (2012). Saliva, salivary gland, and hemolymph collection from *Ixodes scapularis* ticks. J Vis Exp.

[B031] Riihimaa P, Nissi R, Page AP, Winter AD, Keskiaho K, Kivirikko KI (2002). Egg shell collagen formation in *Caenorhabditis elegans* involves a novel prolyl 4-hydroxylase expressed in spermatheca and embryos and possessing many unique properties. J Biol Chem.

[B032] Rice P, Longden I, Bleasby A (2000). EMBOSS: the European Molecular Biology Open Software Suite. Trends Genet.

[B033] Tang X, Cui Y, Namarra U, Tian X, Rivas-Giorgi F, Fikrig E (2024). Dual roles for a tick protein disulfide isomerase during the life cycle of the Lyme disease agent. MBio.

[B034] Tirloni L, Islam MS, Kim TK, Diedrich JK, Yates JR, Pinto AF (2015). Saliva from nymph and adult females of *Haemaphysalis longicornis*: a proteomic study. Parasit Vectors.

[B035] You M, Xuan X, Tsuji N, Kamio T, Igarashi I, Nagasawa H (2001). Molecular characterization of a troponin I-like protein from the hard tick *Haemaphysalis longicornis.*. Insect Biochem Mol Biol.

[B036] Zhang Y, Cui J, Zhou Y, Cao J, Gong H, Zhang H (2018). Liposome mediated double-stranded RNA delivery to silence ribosomal protein P0 in the tick *Rhipicephalus haemaphysaloides.*. Ticks Tick Borne Dis.

[B037] Zhao L, Li J, Cui X, Jia N, Wei J, Xia L (2020). Distribution of *Haemaphysalis longicornis* and associated pathogens: analysis of pooled data from a China field survey and global published data. Lancet Planet Health.

